# Predicting pack-ice seal occupancy of ice floes along the Western Antarctic Peninsula

**DOI:** 10.1371/journal.pone.0311747

**Published:** 2024-12-31

**Authors:** Michael J. Wethington, Bilgecan Şen, Heather J. Lynch

**Affiliations:** 1 Department of Ecology and Evolution, Stony Brook University, Stony Brook, New York, United States of America; 2 Institute for Advanced Computational Science, Stony Brook University, Stony Brook, New York, United States of America; 3 Appalachian Laboratory, University of Maryland Center for Environmental Science, Frostburg, Maryland, United States of America; U.S. Geological Survey, UNITED STATES OF AMERICA

## Abstract

We explore the habitat use of Antarctic pack-ice seals by analyzing their occupancy patterns on pack-ice floes, employing a novel combination of segmented generalized linear regression and fine-scale (∼ 50 cm pixel resolution) sea ice feature extraction in satellite imagery. Our analysis of environmental factors identified ice floe size, fine-scale sea ice concentration and nearby marine topography as significantly correlated with seal haul out abundance. Further analysis between seal abundance and ice floe size identified pronounced shifts in the relationship between the number of seals hauled out and floe size, with a positive relationship up to approximately 50 m^2^ that diminishes for larger floe sizes and largely plateaus after 500 m^2^. These patterns provide information on pack-ice seal behavior and, when combined with methods to delineate individual ice floes, can yield predictions on the number of seals likely to be found in each satellite image scene. This work represents another step in the pipeline required to automate the survey of pack-ice seals using satellite imagery, a necessary step towards pan-Antarctic monitoring of these key marine predators.

## 1 Introduction

Pack-ice seals along the Antarctic Peninsula (crabeater [*Lobodon carcinophaga*], Weddell [*Leptonychotes weddellii*], leopard [*Hydrurga leptonyx*], and Ross seals [*Omnatophoca rossi*]) play an important role as major marine predators in the Southern Ocean (SO) ecosystem, but they are difficult to survey except when hauled out on ice, or, less frequently, land. These seals rely on the sea ice environment for resting, breeding, birthing, and nursing their young, and this habitat also offers access to prey such as krill [1, 2] and protection from predators [3, 4]. As such, the distribution and abundance of pack-ice seals is intimately connected to the nature of the sea ice environment, both because it shapes their movement through the landscape and also because our ability to find seals for the purpose of estimating their abundance is largely dependent on the presence of sea ice as a platform for seals to haul out.

Seal group sizes exhibit significant variability and are likely influenced by a multitude of factors, including seasonal changes, ice cover density, and the availability of food resources. Ribic et al. [5] observed that crabeater seals typically form groups averaging two individuals in areas with dense ice cover, consistent with a more recent study by McMahon et al. [6] in which average group sizes were slightly larger (up to 3.2 seals) and in which it was proposed that the availability of krill in these areas might encourage the formation of larger groups. Further supporting this, Bengtson [7] reported the highest concentrations of crabeater seals in the inner pack ice regions, with groups averaging 1.6 individuals. Bengtson also suggested that the seasonal retreat of ice plays a significant role in aggregating these seals, potentially due to the increased accessibility of krill.

Historically, pack-ice seals have been surveyed using aerial or ship transect surveys (e.g., [8–10]), but this is expensive to do and thus difficult to scale up to large areas. More recently, there has been considerable effort invested in using satellite imagery and computer-vision to automate our survey of seals over large spatial areas [11–14].

Because each seal is small and appears as only a small number of darkened pixels on the ice, convolutional neural networks (CNNs) are most accurate when they take into account the spatial context surrounding the seals in question, and prior work has shown how the probability of detection rises when seals are clustered into groups [11]. As such, correcting estimates of abundance hinges on understanding the statistics of seal group size, yet we have little understanding of how the detailed characteristics of the sea ice environment shape the local density of seals hauled out. As a result, it is difficult to accurately correct satellite-based abundance estimates for detection failures by the CNN.

In this study, we use Very High-Resolution (VHR) satellite imagery to both detect individual pack-ice seals and to examine the sea ice features on which seals are hauled out. This allows us to document the distribution of seal group size across a variety of environmental conditions and, eventually, to differentiate the mechanisms by which broad environmental conditions (e.g., bathymetry, sea ice concentration) influence seal abundance in the water from local-scale drivers of seals hauled out and available for survey. In this study, we focused on pack-ice environments dominated by crabeater seals and while early efforts to identify seal species from satellite imagery appear promising [14], they do not yet allow us to confidently filter out non-target seals from a satellite-based survey. Though we are assuming that the vast majority of seals considered in this analysis are, in fact, crabeater seals, and interpret our findings accordingly, we will use the more generic term ‘seal’ in light of the potential for small numbers of other seal species to be included as well. This work represents the next step in the long-running goal of fully-automated satellite-based seal monitoring in the Southern Ocean, without which it is difficult to validate the efficacy of proposed conservation measures.

## 2 Methods

### 2.1 Data extraction

**Fig 1:**
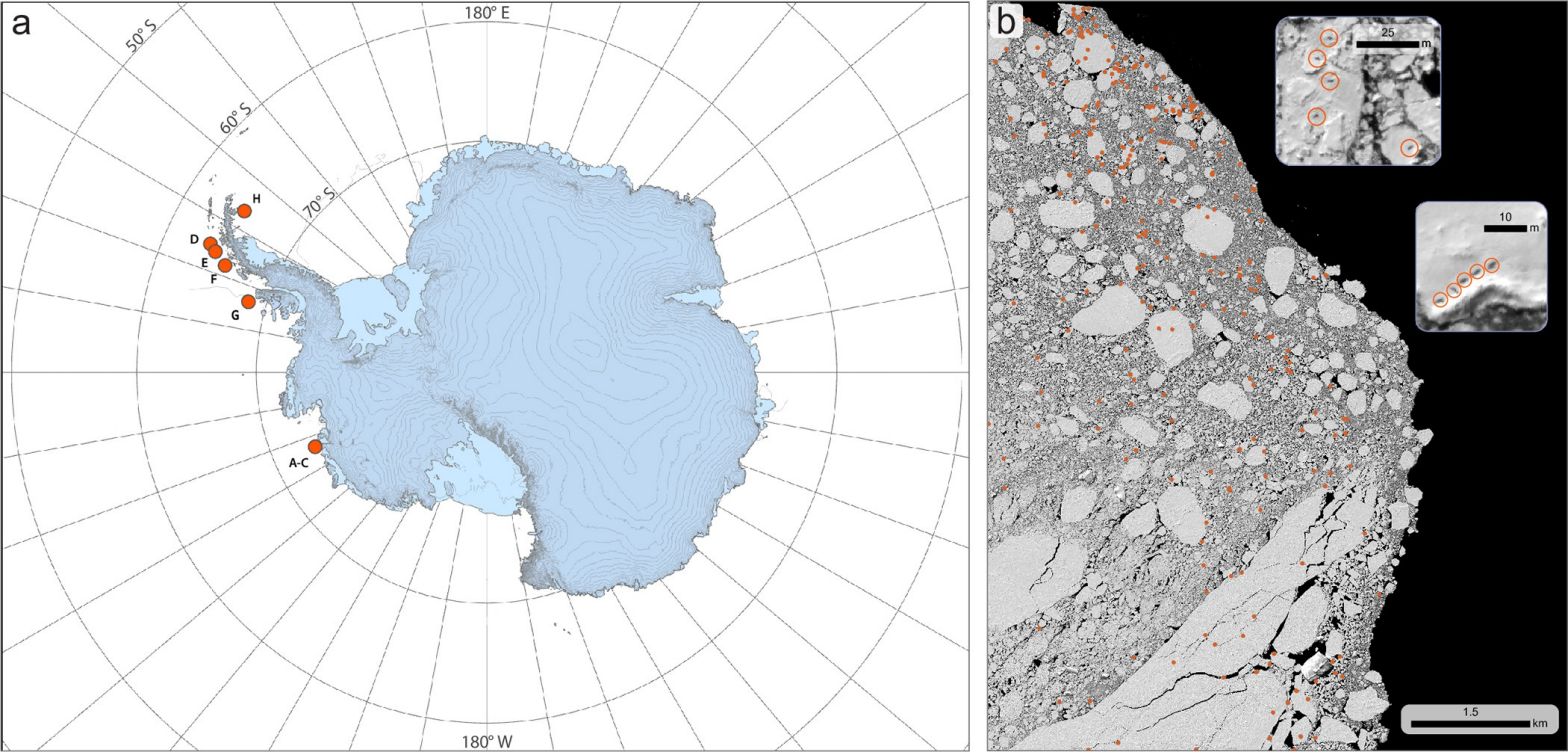
Locations within Antarctica for the scenes used in this study and examples of the pack-ice environment with seal annotations. (a) Locations of the satellite image scenes (pink dots) used in this study. (b) Portion of a satellite image showing the pack-ice environment and the location of individual seals (orange dots). Insets: Annotations of individual seals (orange circles). Figure created in ArcGIS Pro V3.2. Satellite imagery courtesy of Maxar Technologies, provided by the Polar Geospatial Center under the NextView License.

We identified candidate seals in our study using satellite imagery from eight individual panchromatic image scenes, covering a total area of 1,642 km^2^ (Table 1, Fig 1). We selected these images based on two criteria: they had to be cloud-free with minimal shadow interference, and they needed to include a range of ice floe sizes, both occupied and unoccupied by hauled-out seals. To identify candidate seals, we employed the SealNet 2.0 CNN [13] on each image, which initially detected 6,856 seals. Following this, we conducted a comprehensive quality control review by a remote sensing analyst experienced in seal identification from high-resolution satellite imagery. Using a magnification level adequate for distinguishing individual seals, we systematically annotated each image scene to double-check each candidate seal flagged by the CNN and to add any candidate seals not initially identified. This manual review resulted in the addition of 321 seals that were missed by the CNN, bringing the total to 7,177 seals (S1 Table). The overall performance of the SealNet 2.0 ensemble model was evaluated with a precision of 0.80 and a recall of 0.64, indicating that 80% of the identified seals were true positives, and 64% of all seals present were correctly identified. We then cataloged all putative seals in a Geographic Information System (GIS) spatial point database, marking each seal’s location with a geolocated spatial point at its centroid.

**Table 1:** Overview of satellite imagery used in this study.

Label	Scene ID	Platform	Resolution (m)	Area (km^2^)	Cloud Cover (%)	Date
A	1040010016401600 P003	WV03	31	160	0	2015-12-25
B	1040010016401600 P004	WV03	31	160	0	2015-12-25
C	1040010016401600 P005	WV03	31	160	0	2015-12-25
G	10400100196BE200 P010	WV03	31	184	0	2016-02-25
D	1040010035723600 P006	WV03	31	172	0	2017-12-17
E	1040010036AC8000 P004	WV03	31	291	0	2017-12-18
F	1040010058220800 P003	WV03	31	245	0	2020-02-03
H	1040010066439B00 P004	WV03	31	270	0.08	2021-02-09

Scenes A, B, and C overlapped and to avoid potential double counting of seals, we created a mosaic image combining these three scenes and considered them together in our analysis. These scenes are part of the same satellite image strip and were acquired simultaneously. Mosaicing these scenes allowed us to retain more ice floes in the dataset, ensuring that we minimized data loss due to overlaps or the exclusion of floes near the scene edges. This method provided a more accurate and comprehensive dataset for analyzing seal densities. The remaining scenes (D-H) were analyzed individually.

**Fig 2:**
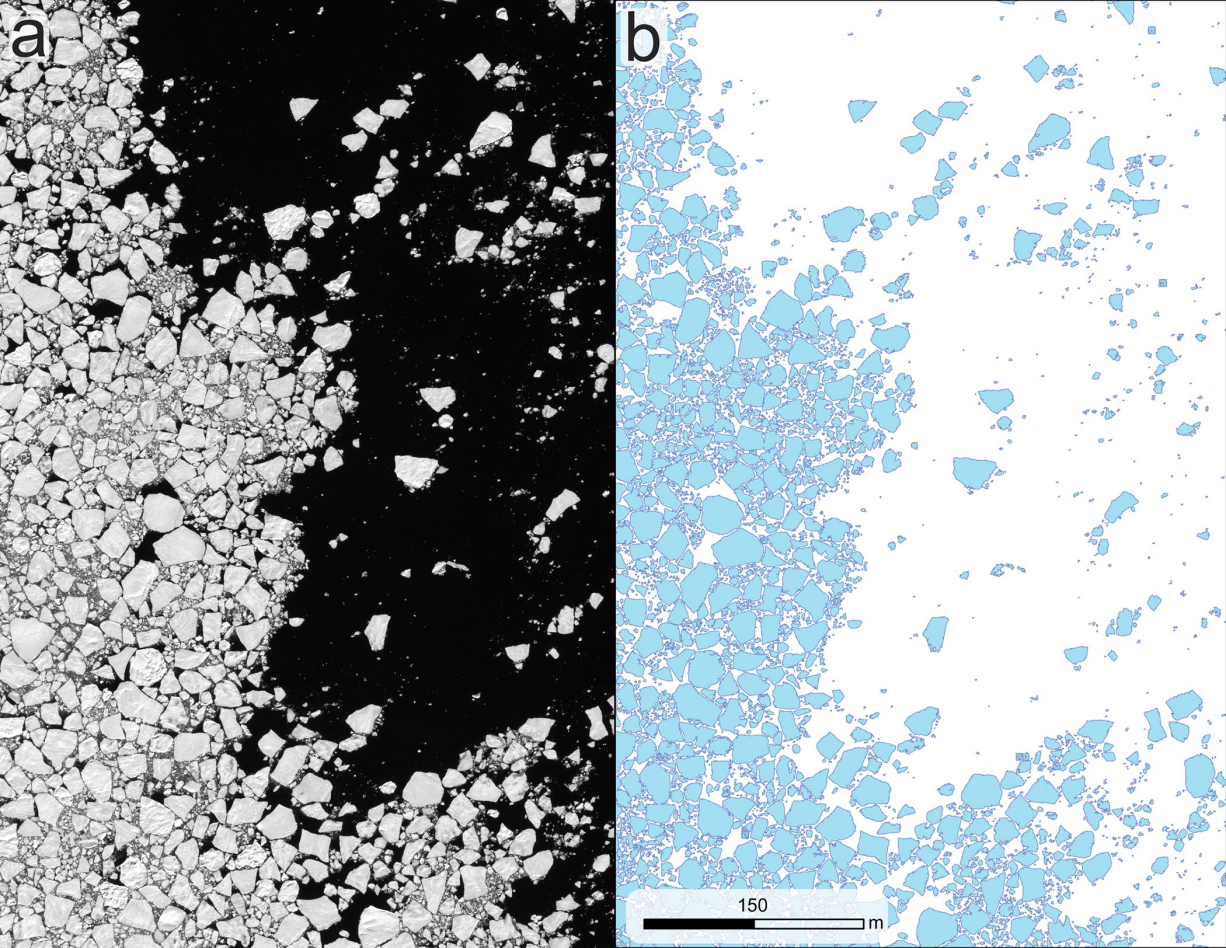
Sea ice segmentation. Input satellite image before sea ice segmentation (a) and individual sea ice floes identified and extracted by CNN segmentation (b). Figure created in ArcGIS Pro V3.2. Satellite imagery courtesy of Maxar Technologies, provided by the Polar Geospatial Center under the NextView License.

We characterized and extracted features of pack-ice (free-floating sea ice) by employing a CNN segmentation process [12] capable of automatically extracting individual ice floes as geospatial polygon features (Fig 2). While the raw resolution of the satellite imagery is 31 cm, the final resolution used for analysis often approximates 50 cm due to resampling during image preprocessing. Two scenes (D and H—see Table 1) exhibited erroneous feature extractions and, to correct errors in the segmentation of floes in these scenes, we applied an object-based image analysis approach on the affected regions in ArcGIS Pro V3.2 [15]. We then merged the corrected sea ice data into scene-specific geospatial polygons and exported them as GeoJSON files.

To examine seal habitat use in relation to nearby environmental features, we fit a statistical model in R [16] incorporating local sea ice concentration (SIC), bathymetric topography characteristics, and proximity to nearby penguin colonies. We used the simple features (sf) [17] and Terra [18] packages to derive all environmental covariates included in our dataset. We calculated SIC at distances of 150, 250, 500, 750, and 1000 meters around each ice floe. We also extracted seafloor bathymetry from the International Bathymetric Chart of the Southern Ocean Version 2 (IBCSO) [19] and used this data layer to compute average (in circles centered on each ice floe) metrics such as bathymetric slope, Terrain Ruggedness Index (TRI), and seafloor roughness.

To avoid artifacts resulting from the image boundary, ice floes situated less than 1000 meters from the edge of the imagery were omitted from the study. This 1000 m buffer was chosen to prevent edge effects because other covariates were considered at radii extending to 1000 m. Importantly, the removal of ice floes and associated seals occurred after the annotation process for seals and ice floes. As a result, this filtering step did not impact seal detection, and only 59 seals were excluded. This exclusion had a minimal effect on the overall dataset, ensuring sufficient statistical power for model fitting and analysis.

The scenes used for this study include over 7.9 million individual ice floes larger than 1 m^2^. We randomly selected 3,000 floes within each of 20 size bins to ensure a complete representation of ice floe sizes in our training dataset. The probability of selecting any given observation was proportionate to the relative frequency of its corresponding size category, thus ensuring an unbiased representation in our sample.

### 2.2 Model feature selection and variable importance

To identify the most influential candidate variables for predicting seal counts, we developed a Conditional Random Forest (RF) model, incorporating the Conditional Permutation Feature Importance (CPFI) metric as outlined by [20]. CPFI diverges from traditional feature importance measures adjusting for the hierarchical structure and interdependencies among variables. This refinement leads to a more precise and impartial evaluation of each predictor’s contribution to the model’s prediction accuracy. By mitigating the bias often associated with correlated predictors in conventional methods, CPFI enables a more reliable assessment of variable importance.

We employed a grid search approach with cross-validation to determine the optimal *ntree* and *mtry* hyperparameters. Model performance evaluation relied on Out-of-Bag (OOB) samples, using Accuracy, RMSE, R-squared, and Mean Absolute Error, and included leave-one-out cross-validation to assess generalizability across different image scenes (see S1 File for more information).

### 2.3 Statistical modelling of seal abundance

The selection process for candidate predictor variables was initially informed by the variable importance rankings derived from our CPFI. High-ranking variables identified as contributing to the model were selected and then passed into the *dredge* function from the *MuMIn* package [21] in R [16] to rank all possible models based on the Akaike Information Criterion (AIC). To mitigate the risk of overfitting associated with this approach, we employed multiple strategies. The top 10 candidate models identified by AIC underwent 5-fold cross-validation to assess predictive performance under different splits of training and testing datasets. This approach ensured that the models were evaluated on unseen data, reducing the likelihood of overfitting. Additionally, the use of multiple performance metrics—Mean Absolute Percentage Error (MAPE) and Root Mean Squared Error (RMSE)—helped to identify models that generalized well across different datasets. Ultimately, the final model was selected using these metrics. We calculated 95% confidence intervals (CI) and prediction intervals (PI) for our final model using the *boot* package [22] in R [16], based on 1000 bootstrap samples of predictions on seal abundance at the scale of each satellite scene. To evaluate the presence of heteroscedasticity and the relationship between model residuals and predicted counts, we used Pearson’s correlation test and White’s test, respectively.

Because CPFI identified ice floe area as the most important variable for predicting the local abundance of seals hauled-out on ice floes, we fit several statistical distributions—Poisson, Negative Binomial, Gamma, Exponential, and Log-normal—to model the number of seals on each ice floe as a function of ice floe area. Because the observed distribution was notably over-dispersed and none of these models provided an adequate fit over the entire range of floe sizes in our dataset, we also explored segmented models using the *segmented* package [23] in R [16] that would allow for the relationship between seal number and floe size to vary across different floe size regimes.

## 3 Results

Our model selection process culminated in the identification of a segmented regression Negative Binomial GLM model as the best fit for our data. This model incorporates four key predictor variables: sea ice floe area (which is treated in a segmented manner), proximity to the nearest penguin colony, bathymetric roughness within 1000 m of the target sea ice floe, and sea ice concentration (SIC) within 750 m of the target ice floe (Table 2). The segmentation of the sea ice floe area allows our model to capture the non-linear effects of this variable on seal counts across different ranges of ice floe sizes.

**Table 2:** Summary of covariates for the final candidate model.

Covariate	Source	Included in final model
Bathymetric roughness at 1000m	IBCSO V2	Yes
Bathymetric slope at 1000m	IBCSO V2	No
Distance to nearest penguin colony	MAPPPD	Yes
Sea ice floe area	CNN extraction	Yes
Sea ice floe perimeter	CNN extraction	No
Sea ice concentration at 750 m	CNN extraction	Yes
Sea ice concentration at 1000 m	CNN extraction	No

Individual variables were included in the final model if they were identified as contributing to model performance.

Segmented regression analysis reveals significant breakpoints in seal count trends at ice floe areas of 49 m^2^ and 481 m^2^. The model demonstrates a consistent, positive relationship between seal count and ice floe area (estimate = 0.139; *p*-value *<*2^*−*16^), albeit with a declining rate of increase beyond each identified breakpoint (Table 3).

**Table 3:** Summary of segmented regression analysis to determine floe area breakpoints at which the relationship between floe area and seal count changes.

Term	Estimate	Std. Error	t-value	p-value
(Intercept)	-7.53	0.18	-41.26	*<*0.001 ***
Area (before Breakpoint 1)	0.139	0.005	30.19	*<*0.001 ***
Area (between Breakpoint 1 and 2)	-0.136	0.005	29.57	NA
Area (after Breakpoint 2)	0.003	1.05 × 10^2^	-26.88	NA
Colony distance	6.19 × 10^*−*7^	3.71 × 10^*−*7^	1.67	0.095
Roughness 1000 m	0.042	0.005	9.06	*<*0.001 ***
SIC 750 m	-0.72	0.04	-18.04	*<*0.001 ***
Breakpoint 1 location	49.84	1.05	N/A	N/A
Breakpoint 2 location	481.32	16.76	N/A	N/A

The Area variable is divided into three segments: before Breakpoint 1, between Breakpoint 1 and Breakpoint 2, and after Breakpoint 2. *Note*: Significance codes: *** p *<* 0.001.

**Fig 3:**
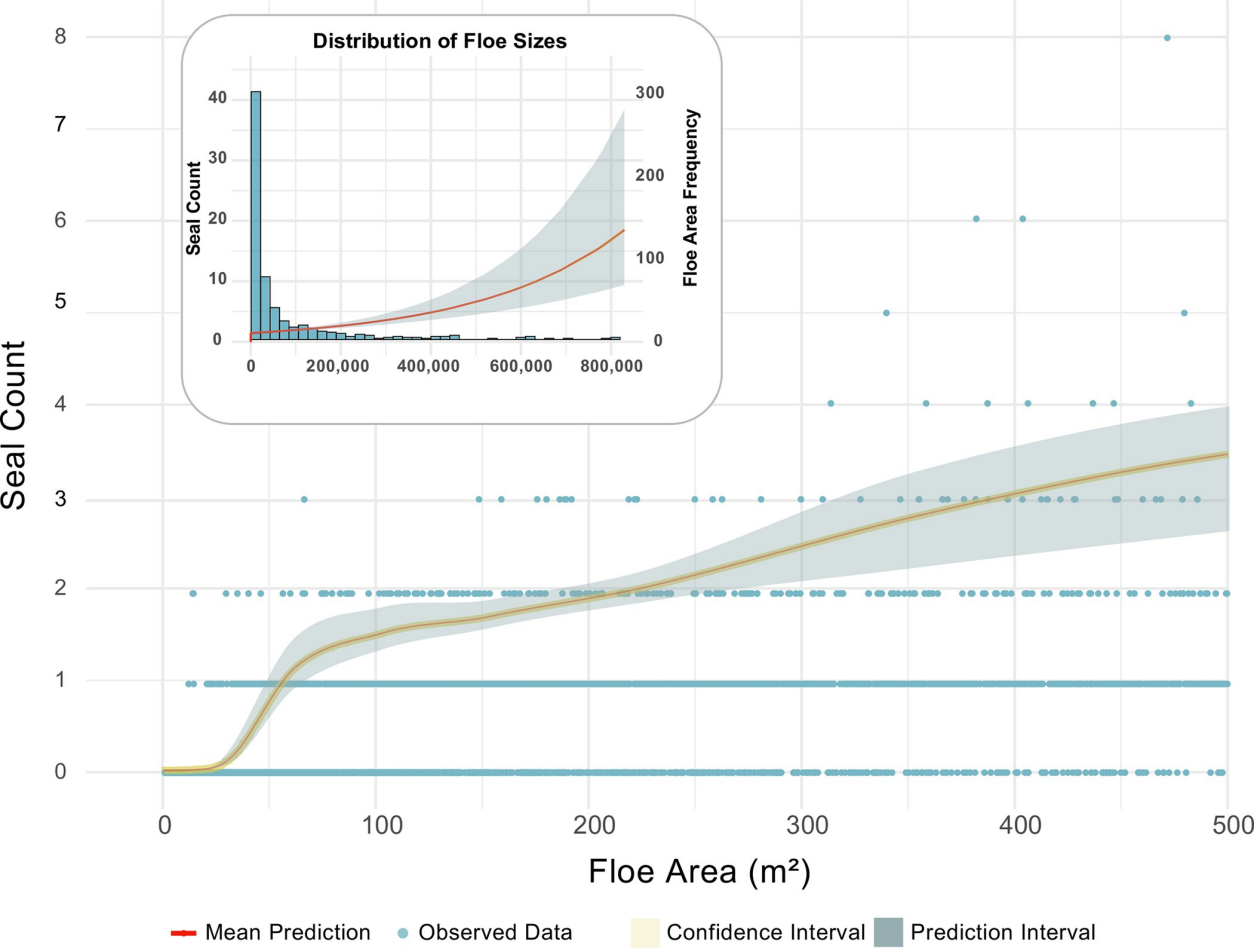
Segmented Generalized Linear Model (GLM) predictions of seal counts for floe areas ranging from 0 to 500 m^2^. The red line represents the mean model prediction, with blue dots indicating observed data. The shaded areas depict the 95% confidence intervals (orange; plotted but too narrow to be visible) and prediction intervals (light grey), detailing the expected range of seal counts. The inset (top left) illustrates the frequency distribution of floe sizes across the entire dataset (x-axis: Floe Area in m^2^; y-axis: Frequency) and includes the mean prediction line with confidence intervals to showcase the model’s performance over a broader range of floe sizes.

Model prediction accuracy varies across different ice floe sizes, with predictions for smaller floes being more precise, as characterized by narrower confidence and prediction intervals (See Fig 3 and S1 and S2 Figs). As ice floe area increases, our predictions tend toward greater uncertainty. Despite this trend, our model demonstrates strong predictive accuracy on a broad scale, evidenced by a dataset-wide Mean Absolute Percentage Error (MAPE) of 11.4% and an average Root Mean Square Error (RMSE) of 0.81 (Table 4 and S2 Table).

**Table 4:** Comparison of observed vs. predicted seal counts across various scenes using a segmented regression Negative Binomial GLM.

Scene	RMSE	MAPE (%)	Observed	Predicted	95% prediction interval
A—C	0.87	10.6	1655	1588	[0.00–47.65]
D	0.41	8.7	965	1131	[0.00–4.17]
E	1.78	17.9	1426	1209	[0.00–56.98]
F	0.34	11.7	1390	1221	[0.00–2.39]
G	0.50	8.6	944	1156	[0.00–4.82]
H	0.82	15.6	797	985	[0.00–2.55]
Overall	0.81	11.38	7177	7290	[0.00–56.98]

Scene-wise RMSE and MAPE metrics, along with total observed and predicted seal counts. “Overall” represents an average across all scenes. Note that scenes A-C were combined as the seals overlapped the three satellite image scenes.

Model performance also varies across individual satellite image scenes used in this study (Table 4, S3 and S4 Tables, and S3 Fig). For instance, in *Scenes A-C* (image mosaic), the model achieves an RMSE of 0.87 and a MAPE of 10.6%, reflecting comparatively high precision. In contrast, *Scene E* displays higher errors with an RMSE of 1.78 and a MAPE of 17.9%, demonstrating the model’s varying performance across different spatial scales. The Pearson’s correlation coefficient between model residuals and predicted seal counts (r = -0.122, 95% CI [-0.136, -0.108], p *<*0.001) suggests a weakly negative correlation and White’s test for heteroscedasticity finds statistically significant variance in residuals across predicted values.

## 4 Discussion

Our model provides reasonably accurate estimates of the number of hauled-out seals on individual sea ice floes, as shown in Table 4. However, predictions at the individual ice floe level exhibit considerable uncertainty, particularly with larger floes (Fig 3). This variability results in both over- and underestimation of local seal counts, depending on the image, with a relative percentage difference of up to ±17.8%. Despite this, since each scene comprises many floes of various sizes, errors tend to offset each other when data are aggregated to broader spatial scales. This leads to more precise abundance estimates at the scene level than at smaller scales (Table 4), as would be expected by the Central Limit Theorem. In the future, such predictions could be incorporated into the loss function of a seal classification model to improve the accuracy of automated seal classification in these complex sea ice environments.

Though abundance estimates at the scene scale were more accurate than at the floe scale, scene-level accuracy did vary across the scenes tested (Table 4, S3 Fig), suggesting the existence of unidentified scene-specific factors (e.g., weather, prey densities, time of day) that might influence the packing density of seals hauling out on ice. Incorporating such scene-level factors will require considerably more replication at the scene-level than was possible in this study given the computational challenges of sea ice segmentation. Given the large number of ice floes per scene, a segmentation algorithm needs to have an exceptionally low error rate to be useful in this context, and we see an improved sea ice segmentation algorithm as the highest priority for future work to fully automate our mapping of Antarctic seals and their sea ice environment.

Ice floe size was the dominant predictor among environmental covariates, overshadowing other factors like ice surface conditions, floe perimeter complexity, and proximity to resource competition. Our results demonstrate a robust positive correlation between the size of ice floes and the number of hauled-out seals. While the positive correlation between ice floe size and seal counts might initially be interpreted as a straightforward carrying capacity effect (i.e. larger floes simply provide more space for seals), the incorporation of additional environmental covariates into the best-predicting model suggests that the preference for larger floes is unlikely to be explained by space availability alone.

Interestingly and somewhat unexpectedly, we identified a significant negative correlation between sea ice concentration (SIC) within a 750 meter radius and seal presence. This suggests that while seals clearly need sea ice to haul out, denser sea ice may discourage them from doing so, possibly because it restricts their physical movement and limits access to open water and haul-out sites. This raises questions about whether dense SIC actually reduces overall seal density in the water column or simply hinders their detection by discouraging seals from hauling out on the ice (where they are visible for satellite-based detection). Disentangling these two mechanisms, which could yield identical censuses of hauled out seals, would require data from tagged seals coupled to nearly-synchronous satellite imagery of the sea ice environment, a capability that is extremely difficult to achieve given current imagery tasking opportunities.

The relationships observed in our study of fine-scale features in the sea ice environment complement other observations on pack-ice seal densities [24–27]. The dependence of crabeater seals, the most numerous pack-ice seal in this region, on ice-dependent krill necessitates a linkage between the abundance of pack-ice seals and concentrated sea ice. Oosthuizen et al. [27] show that at the scale of the most commonly used sea ice products (25 km × 25 km), crabeater seals are found preferentially in areas with high (*>*85%) sea ice concentration. However, the very-high-resolution satellite imagery used in this study allows us to consider how sea ice at much finer resolutions (50 cm vs. 25 km) impacts the number of pack-ice seals hauled out. Here, we find evidence that at the very local scale, high sea ice concentration might actually preclude seals from hauling out. This discrepancy highlights that sea ice influences the abundance of seals in both direct and indirect ways. We must be cautious when extrapolating the influence of sea ice at broad scales, where the mechanism is largely mediated by the abundance of prey, to the direct influence of sea ice at the scale at which seals actually experience their environment. Understanding both pathways is essential if we aim to use very high-resolution satellite imagery (which is unavoidably patchy) to estimate the abundance of pack-ice seals across the Southern Ocean. Local factors will play a crucial role in the probability of detecting seals, as SealNet 2.0 has higher detection rates for groups of seals, and seals must be hauled out on the ice to be detected.

Among the covariates that appears to play a role in the abundance of hauled out seals is local bathymetric roughness, and we saw an apparent affinity for regions where the seafloor presents greater complexity. Burns et al. [28] documented a preference for foraging in locales characterized by steep bathymetric gradients, and Ribic et al. [29] demonstrated a link between the presence of crabeater seals and areas with deep krill concentrations, particularly in bathymetrically diverse regions. Together, these findings underscore the influence of underwater topographical variation on the foraging habits and habitat selection of crabeater seals, highlighting the nuanced interplay between marine geography and ecological behavior.

In addition to identifying areas where pack-ice seals are most likely to be found hauled out on the ice, this study contributes to our understanding of pack-ice seal social behavior. It is well-documented that group sizes vary by species and location, with some individuals remaining solitary and others forming groups of dozens. Average crabeater group size has been estimated between 1.5 to 3.2 individuals but groups as large as 35 have been observed [6]. Our study observed pack-ice seal group sizes ranging from 1 to 44, with an average group size of 1.4 seals per occupied ice floe, broadly consistent with previous work on pack-ice seal group size showing an extreme right skew in the seal count distribution. Such variability appears to be influenced by factors such as local prey availability, the extent of the sea ice landscape, underwater topographic characteristics, and the stability of haul-out platforms [6, 30].

### 4.1 Pack-ice as critical but unprotected habitat

Our study emphasizes the critical role of detailed sea ice data, particularly ice floe size, in accurately predicting seal occupancy and abundance, and underscores the direct physical role that the sea ice environment plays for marine predators such as seals.

Though climate change is poised to have the biggest long-term impact on Antarctica’s sea ice habitat [31], ships moving in and around sea ice during transit and landings can create floes from larger ice sheets and break up larger floes into smaller ones. In this way, vessel traffic, particularly vessels that are deliberately cruising through pack ice, can have an impact on the habitat that pack-ice seals require. Additionally, the noise generated by icebreaking vessels could introduce further disturbances to marine mammals, including seals. Icebreaking operations are far from quiet, and the added noise in this environment may disrupt the behavior of seals and other marine species that rely on acoustics for navigation, communication, and predator detection. Understanding the impact of ship activity, both in terms of physical changes to sea ice and acoustic disturbances, is particularly urgent as the portfolio of tour ships visiting the Antarctic is shifting towards much larger vessels with greater icebreaking capabilities [32]. Whereas tour ships may have traditionally avoided heavily iced areas, the expanded capacity for travel through pack ice presents the risk that ships are changing the sea ice floe environment in ways that are poorly understood with unknown impacts on wildlife, like seals, that require this platform for resting and predator avoidance. Though conservation efforts in the Antarctic have focused on the pelagic zone or on bare-rock terrestrial areas where tourists may be walking around, the pack-ice zone has received considerably less attention and is rarely identified as a critical habitat worthy of monitoring or protection.

These findings of this study collectively highlight the intricate relationship between local sea ice conditions and the ecological behavior of Antarctic pack-ice seals.

Moreover, they underscore the potential for enhancing satellite-based census methods and deepening our understanding of how environmental changes affect these populations. Looking ahead, advancements in computer vision will greatly expand our capacity to identify seals and to characterize the nature of the sea ice environment on which they rely. Our study has highlighted that a large-scale monitoring regime for animals in the pack-ice zone is feasible and provides a clear pathway for a greater ecological understanding and improved conservation in this unique habitat.

### 4.2 Inclusivity in global research

This research was conducted in compliance with ethical, cultural, and scientific standards for inclusivity in global research. Additional information regarding the ethical, cultural, and scientific considerations specific to inclusivity in global research is included in the Supporting Information.

## Supporting information

S1 Fig Plot of residuals vs. predicted counts. (TIF)

S2 Fig Prediction error vs. floe size. (TIF)

S3 Fig Segmented Generalized Linear Model (GLM) predictions of seal counts for floe areas ranging from 0 to 500 m^2^. (TIF)

S1 Table Sea ice floe area and seal count metrics. (DOCX)

S2 Table Averaged model predictions and uncertainty metrics. (DOCX)

S3 Table Summary of model predictions vs. observed data across different scenes. (DOCX)

S4 Table Summary of confidence and prediction intervals for ice floe-level seal counts across different scenes, alongside average observed and predicted seal counts. (DOCX)

S1 Questionnaire Inclusivity in global research. (DOCX)

S1 File (DOCX)
