# Supplementary figures and images for: Predicting pack-ice seal occupancy of ice floes along the Western Antarctic Peninsula
Source: PLoS One. 2024 Dec 31;19(12):e0311747. doi: 10.1371/journal.pone.0311747 (PMC11687692; doi:10.1371/journal.pone.0311747)

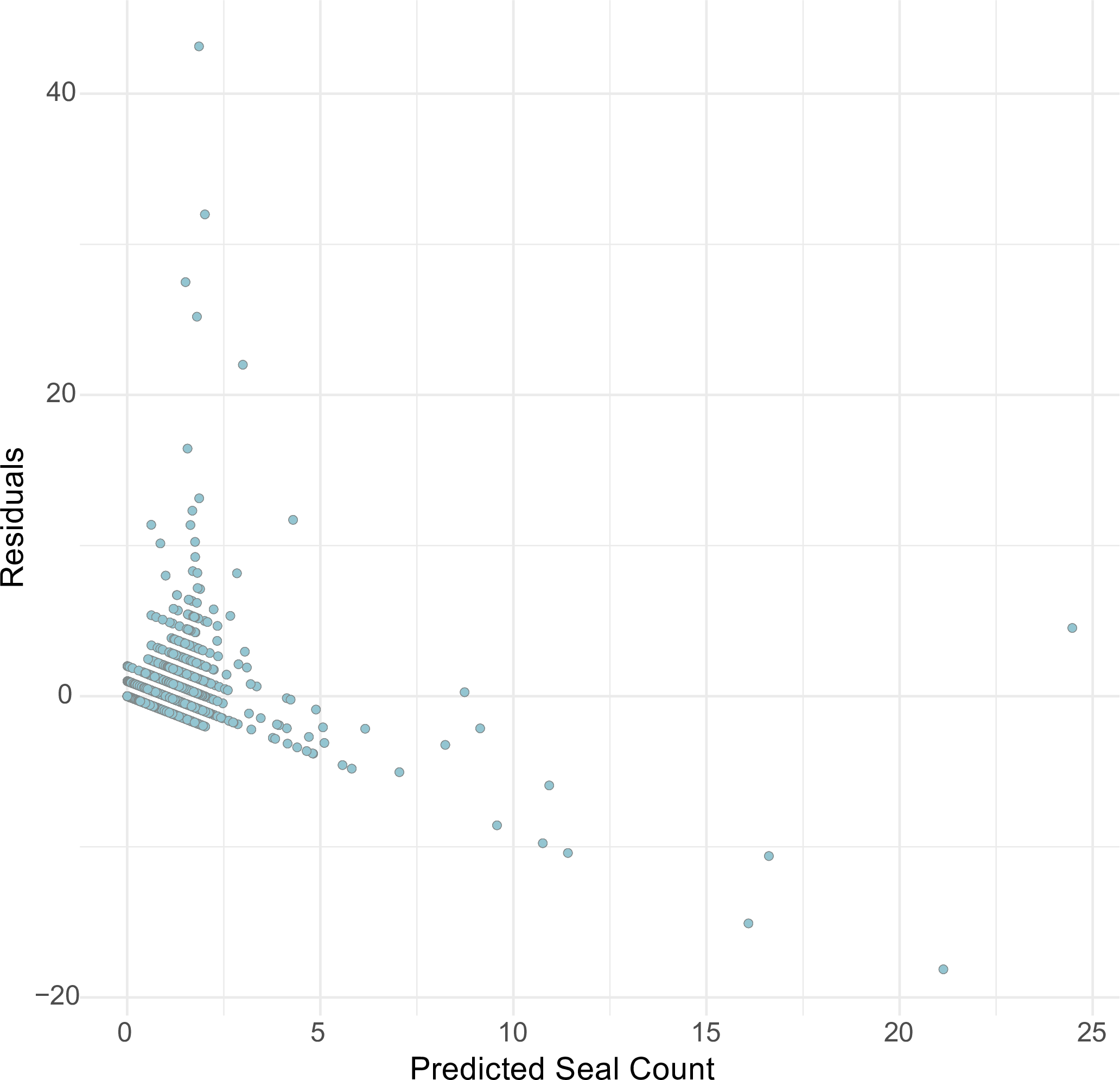

Supplement: S1 Fig — (TIF) [file pone.0311747.s001.tif]

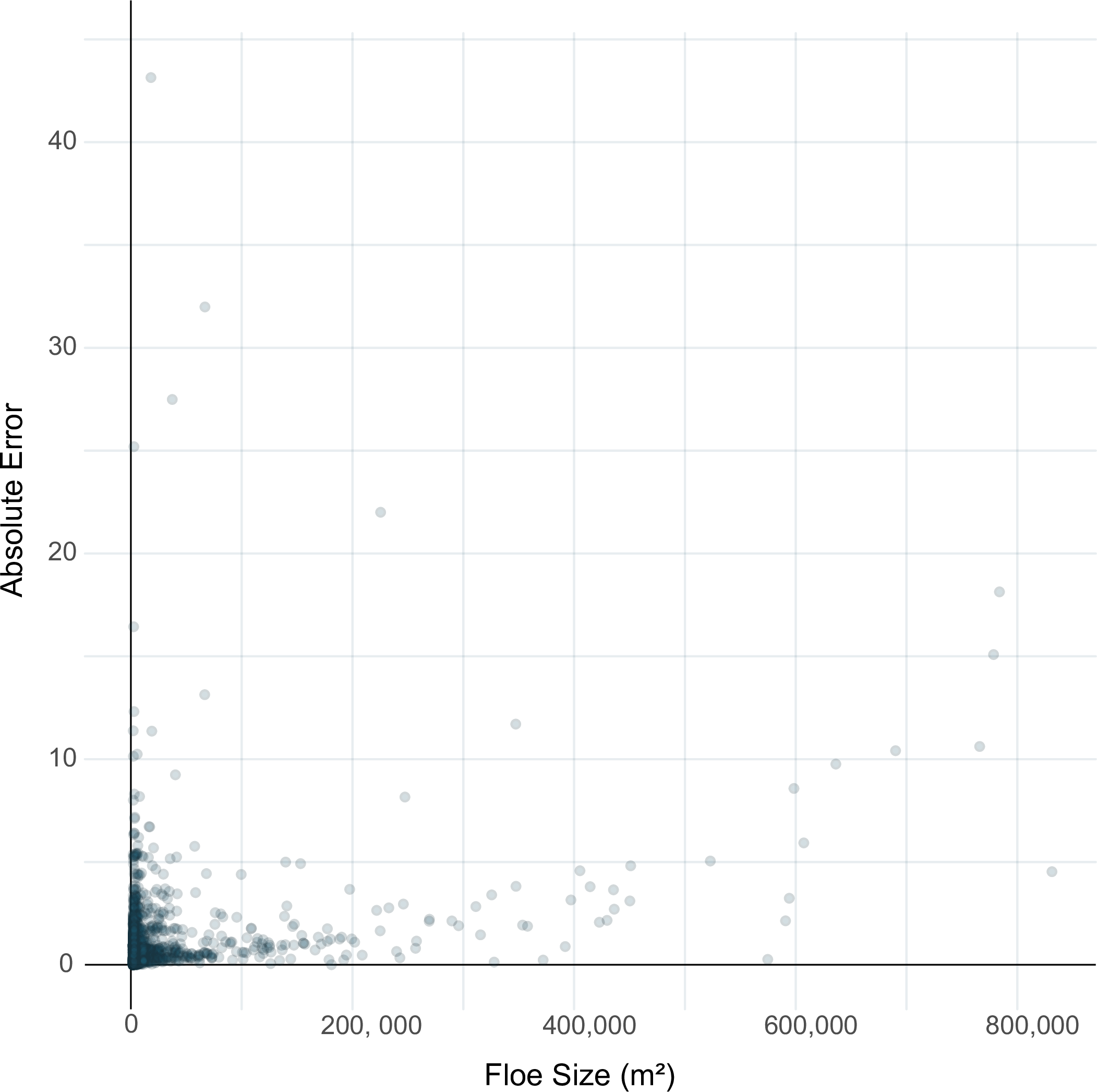

Supplement: S2 Fig — (TIF) [file pone.0311747.s002.tif]

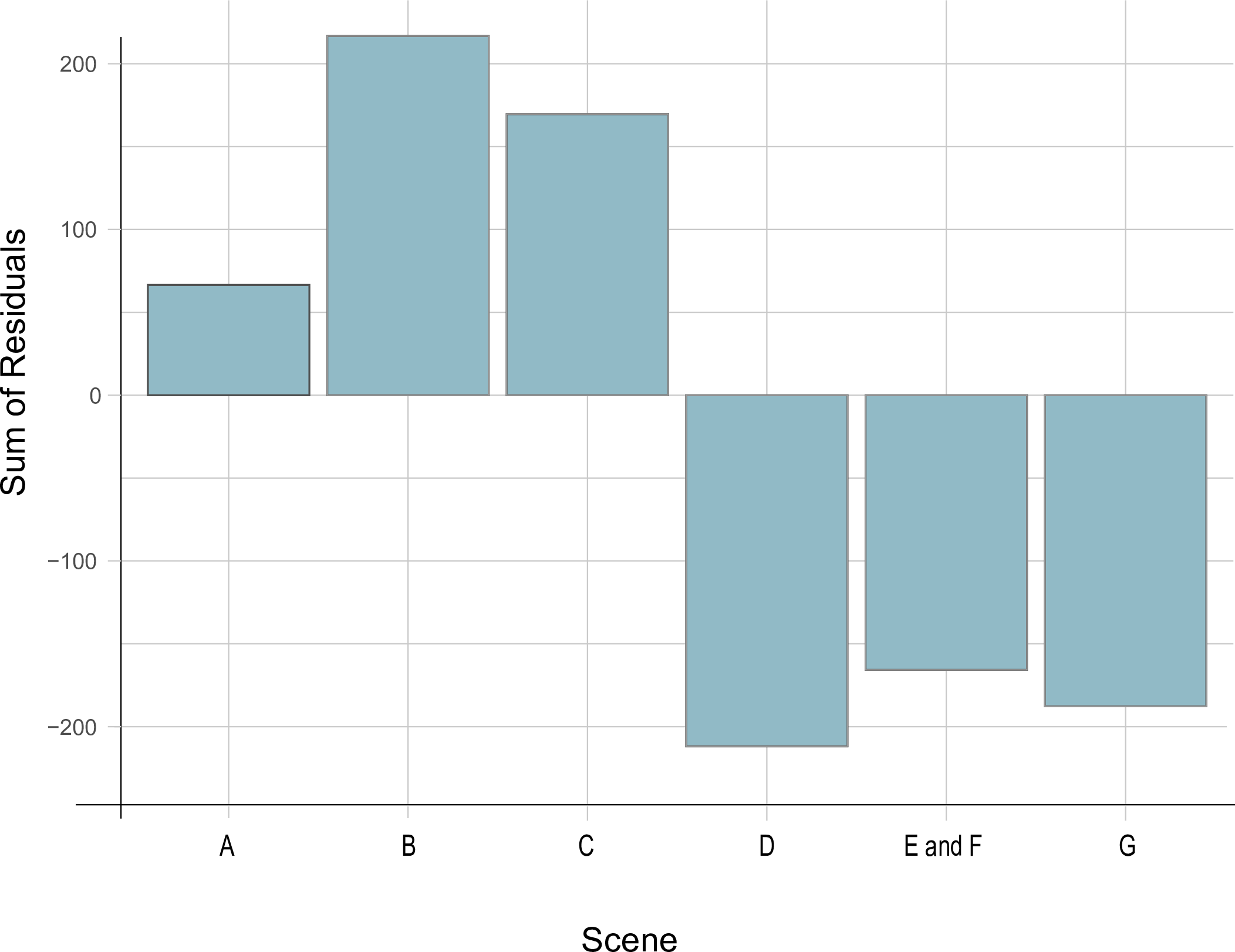

Supplement: S3 Fig — (TIF) [file pone.0311747.s003.tif]
