# Supplementary material for: Predicting pack-ice seal occupancy of ice floes along the Western Antarctic Peninsula
Source: PLoS One. 2024 Dec 31;19(12):e0311747. doi: 10.1371/journal.pone.0311747 (PMC11687692; doi:10.1371/journal.pone.0311747)
Supplement: S1 Table — (DOCX) [file pone.0311747.s004.docx]

**Supplemental Table S1. Sea ice floe area and seal count metrics.**

| Metric | Mean | Median | SD | Min | Max | Q25 – Q75 |
| --- | --- | --- | --- | --- | --- | --- |
| Area (m^2^) | 2263 | 8 | 23534 | 1 | 829263 | 2 – 154 |
| Seal Count | 0.38 | 0 | 1.00 | 0 | 45 | 0 – 1 |
