# Supplementary material for: Predicting pack-ice seal occupancy of ice floes along the Western Antarctic Peninsula
Source: PLoS One. 2024 Dec 31;19(12):e0311747. doi: 10.1371/journal.pone.0311747 (PMC11687692; doi:10.1371/journal.pone.0311747)
Supplement: S2 Table — (DOCX) [file pone.0311747.s005.docx]

**Supplemental Table S2. Averaged model predictions and uncertainty metrics.** This table provides an overall summary of the model’s predicted seal counts per floe across all imagery scenes used in this study. These metrics are calculated without considering the size of the floes, offering a general prediction and its associated uncertainty.

| Metric | Value |
| --- | --- |
| Mean Prediction | 0.382 |
| Prediction SD | 0.701 |
| Prediction Interval (95% PI) | [0.000, 26.076] |
| Lower Prediction Mean | 0.333 |
| Lower Prediction Median | 0.001 |
| Lower Prediction SD | 0.567 |
| Lower Prediction Interval | [0.000, 13.266] |
| Upper Prediction Mean | 0.441 |
| Upper Prediction Median | 0.002 |
| Upper Prediction SD | 0.998 |
| Upper Prediction Interval | [0.001, 56.899] |
| CI Lower Mean | 0.380 |
| CI Upper Mean | 0.384 |
| CI Range | [0.000, 26.803] |
