# Supplementary material for: Predicting pack-ice seal occupancy of ice floes along the Western Antarctic Peninsula
Source: PLoS One. 2024 Dec 31;19(12):e0311747. doi: 10.1371/journal.pone.0311747 (PMC11687692; doi:10.1371/journal.pone.0311747)
Supplement: S3 Table — (DOCX) [file pone.0311747.s006.docx]

**Supplemental Table S3. Summary of model predictions vs. observed data across different scenes.** This table compares the model’s predicted seal counts to the observed seal counts for individual imagery scenes used in this study. The comparisons provide insight to the model’s performance in different contexts

| **Scene** | **Average Predicted Seal Count** | **Average Observed Seal Count** | **SD of Predictions** | **Total Observed Seal Count** | **Total Predicted Seal Count** |
| --- | --- | --- | --- | --- | --- |
| A-C | 0.404 | 0.418 | 0.908 | 1655 | 1598 |
| D | 0.291 | 0.332 | 0.442 | 1390 | 1221 |
| E | 0.588 | 0.693 | 0.925 | 1426 | 1210 |
| F | 0.331 | 0.270 | 0.600 | 944 | 1156 |
| G | 0.313 | 0.266 | 0.585 | 965 | 1131 |
| H | 0.548 | 0.444 | 0.697 | 797 | 985 |
