# Supplementary material for: Predicting pack-ice seal occupancy of ice floes along the Western Antarctic Peninsula
Source: PLoS One. 2024 Dec 31;19(12):e0311747. doi: 10.1371/journal.pone.0311747 (PMC11687692; doi:10.1371/journal.pone.0311747)
Supplement: S4 Table — (DOCX) [file pone.0311747.s007.docx]

**Supplemental Table S4. Summary of confidence and prediction intervals for ice floe level seal counts across different scenes, alongside average observed and predicted seal counts.**

| **Scene** | **Observed** | **Predicted** | **CI** | **PI** |
| --- | --- | --- | --- | --- |
| A-C | 0.418 | 0.404 | [0.40 - 0.41] | [0.35 - 0.49] |
| D | 0.332 | 0.291 | [0.29 - 0.29] | [0.25 - 0.33] |
| E | 0.693 | 0.588 | [0.56 - 0.59] | [0.51 - 0.68] |
| F | 0.270 | 0.331 | [0.33 - 0.33] | [0.30 - 0.37] |
| G | 0.266 | 0.313 | [0.31 - 0.32] | [0.28 - 0.35] |
| H | 0.444 | 0.548 | [0.55 - 0.55] | [0.48 - 0.62] |
