# Supplementary material for: Predicting pack-ice seal occupancy of ice floes along the Western Antarctic Peninsula
Source: PLoS One. 2024 Dec 31;19(12):e0311747. doi: 10.1371/journal.pone.0311747 (PMC11687692; doi:10.1371/journal.pone.0311747)
Supplement: S1 File — (DOCX) [file pone.0311747.s009.docx]

**Supplementary Material for Predicting pack-ice seal occupancy of ice floes along the**

**Western Antarctic Peninsula**

**——**

Michael Wethington^1^, Bilgecan S¸en^1,2,3^, and Heather J. Lynch ^1,2^

^1^Department of Ecology and Evolution, Stony Brook University

^2^ Institute for Advanced Computational Science, Stony Brook University, Stony Brook, New York, USA

^3^Appalachian Laboratory, University of Maryland Center for Environmental Science, Frostburg, Maryland, United States of America

**Supplemental Table S1. Sea ice floe area and seal count metrics.**

**Supplemental Table 2. Averaged model predictions and uncertainty metrics.** This table provides an overall summary of the model’s predicted seal counts per floe across all imagery scenes used in this study. These metrics are calculated without considering the size of the floes, offering a general prediction and its associated uncertainty.

**Supplemental Table S3. Summary of model predictions vs. observed data across different scenes.** This table compares the model’s predicted seal counts to the observed seal counts for individual imagery scenes used in this study. The comparisons provide insight to the model’s performance in different contexts

**Supplemental Table S4. Summary of confidence and prediction intervals for ice floe level seal counts across different scenes, alongside average observed and predicted seal counts.**

1. **Variable importance using random forest models**

To evaluate the relative importance of covariates in our dataset, we developed a Random Forest (RF) model, following the methodologies established by Breiman [[2]](#_bookmark35) and expanded upon by Cutler et al. [[3].](#_bookmark36) RF models are robust against common pitfalls such as overfitting, which often plagues traditional decision trees. This robustness stems from an ensemble learning approach that proves particularly effective when analyzing datasets with numerous potential covariates. Key adjustable hyperparameters in RF models include *ntree* and *mtry*, which are essential for optimizing the model’s configuration.

We chose the conditional inference RF model variant as per Strobl et al. [[20]](#_bookmark24) due to its efficacy in managing interrelated variables and providing accurate estimates of variable importance. This variant differs from traditional RF models by utilizing a non-biased subsampling technique without replacement, which is ideal for datasets featuring complex variable interactions.

For the model’s implementation, we employed the *cForest* function from the Party package [[4]](#_bookmark37) in R, with hyperparameter selection guided by a grid search optimized through cross-validation using the Caret package [[5].](#_bookmark38) We conducted the search with a ten-fold cross-validation strategy to evaluate of the model’s performance across various data segments. The hyperparameters were set at *ntree* = 7,500 and *mtry* = 12.

- 1. **Assessing variable importance**

To assess variable importance, we used the RF Conditional Permutation Feature Importance technique, described by Strobl et al. [[1],](#_bookmark24) which rearranges each feature

Figure_S1.pdf

**Supplemental Figure S1.** Residuals vs. predicted counts illustrating the heteroscedastic distribution of residuals against predicted counts, with a funnel-shaped spread indicating underestimation at lower predictions and overestimation at higher predictions. Notable outliers at lower predicted counts suggest the presence of potential anomalies or inaccuracies in the model’s predictions.

Figure_S2.pdf

**Supplemental Figure S2. Prediction Error vs. Floe Size**

Figure_S3.pdf

**Supplemental Figure S3.** Error Residuals by Scene

among observations with similar values of other predictors. This approach preserves the correlation structure among features and variables, providing a more nuanced evaluation than traditional methods like Gini Impurity or basic permutation feature importance.

This technique is computationally demanding but offers significant advantages. It not only provides more reliable feature importance scores by considering feature interactions but also enables assessment of individual feature importance rather than just relative importance. It is particularly robust against correlated features, a critical aspect since covariates at nested radii are inherently correlated. Moreover, it measures feature importance in terms of actual changes in model accuracy, enhancing interpretability [[1,](#_bookmark24) [2,](#_bookmark35) [4,](#_bookmark39) [5].](#_bookmark40)

1. **RF model performance evaluation**

We evaluated the performance of our Random Forest models using the Out-of-Bag (OOB) sampling method. This method involves using the entire dataset for training, which contrasts with conventional approaches that set aside part of the data for testing. As such, we could leverage all available seal location data to robustly estimate model performance without requiring a separate test set.

For performance metrics, we specifically selected RMSE, R-squared (R^2^), and MAE due to their relevance to our model, which predicts seal abundance based on occupancy data:

- RMSE (Root Mean Square Error) offers insights into the accuracy of predictions by measuring the standard deviation of the prediction errors.
- R^2^ indicates the proportion of variance in seal counts explained by the model, with values closer to 1 indicating high explanatory power.
- MAE (Mean Absolute Error) measures the average magnitude of errors, providing a direct interpretation of prediction accuracy.

To ensure the model’s reliability across various scenarios, we employed a

leave-one-out cross-validation strategy, reserving each scene in turn as the test set while training on the remaining data. This method showed that our performance metrics were

consistent with those from full-dataset training, underscoring our model’s robustness and generalizability.

- 1. **Random forest variable importance results**

Our analysis revealed that the most significant predictor of seal presence and quantity is the sea ice floe area, with a relative importance score of 0.993. Lesser but still notable influences include sea ice concentration (SIC) within 150 meters of an occupied ice floe and the perimeter of the ice floe, with importance scores around 0.006 and 0.005, respectively. These findings indicate that both the conditions and complexity of the ice surface play roles in predicting seal occupancy and abundance, albeit to a lesser extent than floe area.

The proximity to penguin colonies and various terrain features across scales showed minimal predictive power. Interestingly, some variables such as mean terrain ruggedness and certain SIC measurements exhibited negative importance values, suggesting they might reduce model accuracy due to multicollinearity, especially when correlated with more significant predictors like ice floe area.

- - 1. Strobl C, Boulesteix AL, Kneib T, Augustin T, Zeileis A. Conditional variable importance for random forests. BMC bioinformatics. 2008;9:1–11. Available from: <https://doi.org/10.1186/1471-2105-9-307>.
    2. Breiman L. Random forests. Machine learning. 2001;45(1):5–32. Available from:

https://doi.org/10.1023/A:1010933404324.

- - 1. Cutler DR, Edwards Jr TC, Beard KH, Cutler A, Hess KT, Gibson J, et al. Random forests for classification in ecology. Ecology. 2007;88(11):2783–2792. Available from: <https://doi.org/10.1890/07-0539.1>.
    2. Hothorn T, Hornik K, Strobl C, Zeileis A, Hothorn MT. Package ‘party’. Package Reference Manual for Party Version 09-998. 2015;16:37. Available from: [https://CRAN.R-project.org/package=party](https://CRAN.R-project.org/package%3Dparty).
    3. Kuhn M. The caret package. R Foundation for Statistical Computing, Vienna, Austria. 2012;Available from: [https://cran.r-project.org/package=caret](https://cran.r-project.org/package%3Dcaret).
    4. Altman NS. An introduction to kernel and nearest-neighbor nonparametric regression. The American Statistician. 1992;46(3):175–185. Available from: <https://www.tandfonline.com/doi/abs/10.1080/00031305.1992.10475879>.
    5. Hastie T, Tibshirani R, Friedman JH, Friedman JH. The Elements of Statistical Learning: Data Mining, Inference, and Prediction. vol. 2. Springer; 2009. Available from: <https://doi.org/10.1007/978-0-387-21606-5>.
